# Expanded Utility of Human Acellular Vessel in Hemodialysis Access Surgery and Arterial Aneurysm Repair

**DOI:** 10.7759/cureus.46325

**Published:** 2023-10-01

**Authors:** Margaret C Nickerson, Aish Thamba, Varun Rao, David B Peterson, David A Peterson, Duangnapa S Cuddy

**Affiliations:** 1 Department of Vascular Surgery, Indiana University School of Medicine, Bloomington, USA; 2 Department of Vascular Surgery, Indiana University School of Medicine, IU Health Bloomington Hospital, Bloomington, USA

**Keywords:** arteriovenous access, end-stage renal disease, aneurysm repair, regenerative medicine advanced therapy, human acellular vessel, vascular access, hemodialysis

## Abstract

Vascular access is essential for hemodialysis (HD) in patients with end-stage renal disease (ESRD). When the standard of care arteriovenous fistula (AVF) is limited, secondary to aneurysmal degeneration, trauma, and thrombus, interposition grafting is a reasonable reconstruction approach. As these grafts and comorbidities place ESRD patients at sustained risk of complications, reconstructions with regenerative medicine biologic conduits hold promise in improving safety and efficacy. Here, a biocompatible human acellular vessel (HAV) is our conduit of interest. With United States Food and Drug Administration use authorization under the Expanded Access Program, we report three cases of complex vascular access surgery with four aneurysm repairs using HAV. Patient selection focused on meeting unmet needs for those without adequate care alternatives, including active access and endoprosthetic stent graft infections, right heart failure due to high-output AVF, and arterial and access outflow aneurysms. In this high-risk expanded access population, operative technical success and interval success for patients given their inherent comorbidities, offer potential expanded utility of HAV in HD access surgery and arterial aneurysm repair.

## Introduction

Functioning vascular access is essential for hemodialysis (HD) in patients with end-stage renal disease (ESRD). While arteriovenous fistula (AVF) is the preferred method for long-term vascular access, the prevalence of aneurysmal degeneration may be high [1]. True aneurysm and pseudoaneurysm rates may vary from 5% to over 60% because of the failure to recognize aneurysms. Aneurysms pose a significant risk to the patient and their continued vascular access for HD, including rupture, pseudoaneurysm and hematoma formation, bleeding, abnormal access flow, infection, thrombosis, and stenosis [2].

Dialysis access grafts are appropriate options for HD if ESRD patients are not suitable candidates for AVF or if AVFs are no longer functional despite percutaneous intervention [3,4]. However, synthetic expanded polytetrafluoroethylene (ePTFE) arteriovenous grafts (AVG) may be associated with infection rates of up to 9% per patient year and secondary patency of only 70% at one year [5]. Xenografts and allografts may have similar complications impacting graft patency, including dilatation, stenosis, aneurysm, and host immune response [5,6].

In 2016, the United States Food and Drug Administration (FDA) 21st Century Cures Act established the Regenerative Medicine Advanced Therapy (RMAT) designation, granted as individual investigational new drugs (INDs). The first Expanded Access Program (EAP) in Bloomington, Indiana, supports the compassionate use of the biocompatible human acellular vessel (HAV) (Humacyte, Inc., Durham, NC, USA) as an expanded utility vascular surgical conduit for use in ESRD, peripheral arterial disease (PAD), and vascular trauma [7-9].

Bioengineered in a bioreactor, HAV is an investigational, shelf-stable human tissue-engineered vascular conduit, which is decellularized to mitigate implant rejection [6]. HAV has been used in clinical trials as an HD vascular access conduit in ESRD and for vascular reconstruction in PAD and vascular trauma [3,4,6,10,11]. Most recently, HAV has been utilized for humanitarian vascular trauma repair in Ukraine, and enrollment was completed for a Phase II/III vascular trauma trial (V005) to further investigate the use of human acellular vessel (HAV) in vascular trauma repair [12]. This biologic conduit populated with patient cells may better resist infection [4,6,13]. Infection rates were 1.3% per patient year in two phase 2 single-arm trials, and conduits displayed improvement in structural integrity and patency as secondary patency was observed to be 90% at 1 year. This makes HAV a beneficial option for patients with complicated HD vascular access situations, including occlusion, thrombosis, and aneurysm [4,6,13].

This case series includes patients with life-threatening conditions, multiple comorbidities, and limited options for vascular reconstruction in compassionate use cases starting December 2021. Participants were unsuitable for a standard ePTFE graft and would not meet the criteria for existing clinical trials, necessitating a conduit for maintaining HD access made available through the EAP. Written informed consent for patient information was provided by the patients. We are sharing intermediate experiences and postoperative outcomes from the first year of the EAP. We aim to share these individuals’ experiences and outcomes with EAP and RMAT, respectively, in our study. Ongoing patient follow-up protocol after RMAT has been conducted, including optimizing medical therapy, reducing risk factors, and reporting postoperative courses as per FDA guidelines. The Indiana University IRB approved all EAP care and manuscript scope (IRB 13420, 18041).

## Case presentation

Case 1

A 48-year-old woman on HD for ESRD with a history of lupus anticoagulant syndrome, diabetes mellitus type II, COVID-19 pneumonia, and left-finger osteomyelitis presented with partially aneurysmal thrombosis of her left brachiocephalic AVF placed three years prior. The AV anastomosis and a short segment of inflow cephalic venous fistula within 6 cm of the anastomosis appeared to be patent. History is notable for previously placed stent in the outflow cephalic venous fistula. The cephalic venous outflow limb beyond the aneurysmal segment was well patent. The preoperative plan was inline conduit reconstruction bypassing the aneurysm, with inflow from the proximal healthy stump of the distal cephalic vein 5 cm from the arterial anastomosis and outflow from the proximal cephalic vein beyond the stent using the HAV conduit, along with removal of the existing stent.

**Figure 1:**
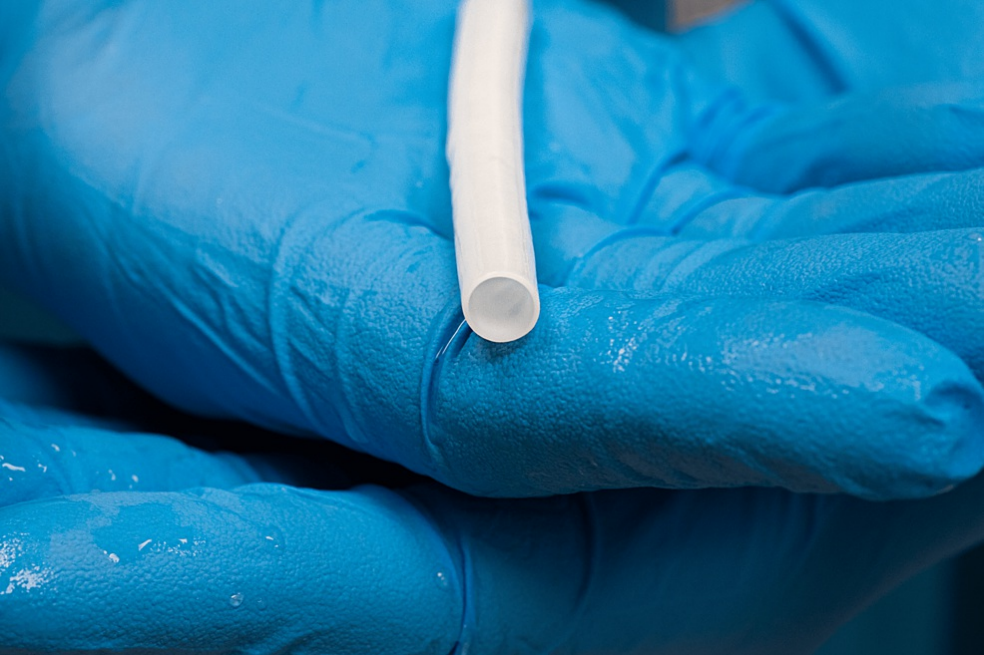
Human Acellular Vessel Shared with courtesy and manufacturer permission.

The HAV conduit (0.6 cm x 40 cm) (Figure 1) was prepared to manufacturing standards. The conduit is stored in a packaging system with a sterile phosphate buffer solution and refrigerated at 4-8 degrees Celsius. No thawing is necessary, and the conduit can be stored at room temperature for up to 48 hours prior to use. Intraoperatively, the sterile buffer solution was drained, and the conduit was removed from its packaging system. The inner mandrel was removed from the conduit, which was flushed with heparinized saline and then prepared for anastomosis using standard techniques.

The interposition graft was anastomosed in an end-to-end fashion to the distal cephalic venous fistula stump as the inflow conduit and in an end-to-end fashion to the proximal cephalic venous fistula stump beyond the area of the cephalic venous outflow stent placement. Flow was visualized with intraoperative HD access duplex ultrasound, which demonstrated no evidence of obstruction or complications throughout the conduit and in the distal brachial, radial, and ulnar arteries. Flow was found to be 994 mL/min in the proximal segment and 1003 cc/min midsegment. Additionally, the existing stent was explanted, and the left arm AVF aneurysm was resected and ligated. The patient tolerated the operation well and was discharged home the same day.

The HAV conduit served as HD access within four weeks. At the six-week follow-up, the conduit was patent with volume flow measuring 793 mL/min, velocities within normal limits, and no areas of significant diameter reduction noted. Primary patency was maintained at the patient’s three-month follow-up. Four months after the surgery, the patient was admitted to the hospital for cardiogenic shock and hyperkalemia, unrelated to the HAV reconstruction procedure. She developed pericardial effusion and cardiac tamponade and transitioned to palliative care.

Case 2

A 44-year-old man on HD for ESRD with a history of congestive heart failure, chronic obstructive pulmonary disease, and tobacco use disorder presented with a >5 L/min left AVF aneurysm requiring a semi-urgent procedure. The preoperative goal was to resolve high-output heart failure and reestablish HD access using HAV conduit anastomosis and resection of the AVF aneurysm.

However, during the operation, two aneurysms were encountered, including the brachial artery (3 cm) and the majority of the brachiocephalic fistula outflow tract (4 cm). The distorted anatomy of the patient mandated a brachial artery reconstruction with HAV, permitting ligation of the AVF to treat high-output heart failure. This was conducted by a series of regenerative reconstruction (artery-HAV-artery end-to-end interposition repair) of the brachial artery aneurysm, followed by proximal and distal ligation of his disability-creating AVF outflow tract. The patient's standard postoperative course included discharge to home the subsequent day.

As the HAV was used for brachial artery reconstruction, it was not utilized as vascular access for HD. Two months after surgery, the patient presented to dialysis with substernal chest pain and ST elevations on EKG. An infection was localized to his HD catheter, necessitating the removal of his internal jugular catheter. He was found to have methicillin-resistant Staphylococcus aureus infective endocarditis of the aortic and mitral valves and developed septic shock. The patient was not a surgical candidate and was transitioned to palliative care.

Case 3

A 66-year-old woman requiring HD for ESRD presented with a history of three-year-old left radiocephalic AVF with a prior balloon angioplasty intervention, diabetes mellitus type II, severe obesity, multiple venous thromboembolic events requiring ongoing anticoagulation, and limited mobility. In 2022, she had a bleeding skin ulcer from the infected pseudoaneurysm of her HD access and urgently underwent covered stent repair to control catastrophic bleeding. HD access modality was changed to a tunneled dialysis catheter. To restore functionality of the existing HD access, the preoperative plan was venous-venous HAV interposition conduit to left arm AVF, along with resection of the endoprosthetic stent graft and AVF aneurysmal segment. A surgical technique with an interposition biological conduit was chosen to potentially reduce infection risk due to acutely infected anatomy in the operating field.

As a planned stage operation, the HAV conduit was prepared to manufacturing standards and was then anastomosed in an end-to-end fashion to the inflow cephalic venous fistula stump. Then, it was anastomosed to the outflow cephalic venous fistula stump in an end-to-end fashion. Due to the infected operative field, the infected AVF inclusive of the stent along with the overlying subfascial infected skin was removed. Volume flow across the HAV conduit was recorded to be between 480-500 mL/min via intraoperative hemodialysis access duplex ultrasound.

The patient had no postoperative complications. At a four-week follow-up, volume flow was borderline normal at 486 mL/min, with velocities within normal limits and no evidence of conduit stenosis. A remnant of the radiocephalic AVF outflow was increased in diameter from 0.4 cm to 0.84 cm at the HAV conduit inflow. The dilated segment measured 0.84 cm x 0.98 cm in length. Patency, adequate cannulation, and HD access via HAV were established, and the dialysis catheter was removed. At the three-month follow-up, flow was noted to be within normal limits at 600 mL/min. The patient’s HAV maintained primary patency with active HD access for five months. The patient’s treatment for thrombophilia is ongoing per hematology, and she continued her established anticoagulation regimen of 5 mg apixaban twice daily postoperatively. Of note, HAV implantation requires postoperative antiplatelet therapy as with any reconstruction or revascularization procedure but does not require anticoagulation in the absence of indications for the patient as in this case. Due to her recalcitrant thrombophilia, her conduit thrombosed, and she underwent a technically successful secondary intervention via transcatheter thrombectomy and venous outflow angioplasty with a 5x60 mm drug-coated balloon. Total conduit patency was about six months. The patient currently undergoes catheter-dependent hemodialysis.

## Discussion

HD vascular access complications are associated with high morbidity and mortality, highlighting a critical need for novel techniques in establishing and maintaining adequate vascular access [3]. The patients in this study underwent implantation with an HAV conduit due to intrinsic challenging characteristics that made them candidates for the novel HAV approach for aneurysm repair. These patients were high risk and unable to qualify for clinical trials for alternative methods, according to FDA qualifications for the EAP and specifications for existing clinical trials. The patient in Case 1 had several preexisting partially aneurysmal thromboses, creating an anatomically challenging landscape not inherently functional for HD access. Venogram and fistulogram determined adequate patent inflow and outflow patency for interposition revision, compatible with the regenerative approach. In Case 2, the patient had a prominent AVF aneurysm thatresulted in degenerative configurational changes of the brachiocephalic venous fistula, leading to a severely distorted and challenging anatomical environment to identify the true anastomosis of the brachiocephalic AV fistula. In Case 3, the patient experienced an adverse event of infection of her HD site secondary to skin ulceration in the pseudoaneurysm overlying the HD access site, with emergent bleeding complications. These patients, as is true of all patients with ESRD, also had multiple comorbidities, including obesity, type 2 diabetes, congestive heart failure, and thromboembolism events requiring prompt operations to maintain HD access (Table 1).

**Table 1:** Patient Characteristics and Postoperative Outcomes

Case #	Age (years)	Comorbid Conditions	Notable Preoperative Features	Surgical Procedure	Postoperative Outcomes
1	48	End-stage renal disease, lupus anticoagulant syndrome, diabetes mellitus type II, COVID-19 pneumonia, left finger osteomyelitis.	Aneurysmal thrombosis of left brachiocephalic AVF with prior stent placement.	Regenerative reconstruction (vein-HAV conduit-vein end-to-end interposition repair) of brachiocephalic AVF aneurysm. Stent removal and AVF aneurysm ligation.	No postoperative complications. HD conduit for three months with primary conduit patency. Patient passed away from complications of pericarditis and sepsis, unrelated to HAV reconstruction procedure.
2	44	End-stage renal disease, congestive heart failure, chronic obstructive pulmonary disease, tobacco use disorder.	Left brachial artery aneurysm and high-output (>5L/ minute) brachiocephalic AVF aneurysm.	Regenerative reconstruction (artery-HAV conduit-artery end-to-end interposition repair) of brachial artery aneurysm. Proximal and distal ligation of AVF aneurysm.	No postoperative complications. The patient passed away two months later due to complications of MRSA endocarditis and heart failure likely unrelated to the procedure.
3	66	End-stage renal disease, diabetes mellitus type II, obesity, multiple venous thromboembolic events.	Bleeding, infected pseudoaneurysm of three-year-old left radiocephalic AVF with prior balloon angioplasty procedure.	Regenerative reconstruction (vein-HAV conduit-vein end-to-end interposition repair) of radiocephalic AVF aneurysm. Stent and AVF aneurysm removal.	Adequate cannulation, conduit patency, and HD access establishment for five months. Transcatheter thrombectomy and outflow vein angioplasty then performed due to recalcitrant thrombophilia and small outflow venous diameter.

Following HAV conduit placement, Cases 1 and 2 resulted in successful repair of their respective aneurysms and, therefore, maintained conduit patency until they succumbed to unrelated sequelae. Case 1 was able to maintain HD access with HAV for four months. Case 3 resulted in similarly technically successful aneurysm repair and maintained HD access for five months until the conduit thrombosed, as previously mentioned. Of note, the placement of the biologic HAV conduit in each patient displayed infection tolerance without heightened immune response or aneurysmal degeneration. In comparison, ePTFE grafts have high rates of loss of primary unassisted patency and long-term patency in patients, 75% within one year, and 27% within five years. This leads to the placement of central venous catheters for HD sites, which are associated with increased rates of adverse events such as all-cause mortality, bacteremia, and vascular access thrombosis [13]. Furthermore, HAV remodeling postoperatively has shown microvessel remodeling with cells that mimic native blood vessel composition and demonstrate continued durability and patency with a low risk of infection [11]. The HAV conduit has proven to be advantageous in another two phase 2 single-arm trials which showed reduced post-cannulation bleeding, immune response, mechanical failure, and incidence of adverse events, mimicking our progress thus far [4]. In the present series, we utilized the HAV conduit resulting in the technical success of regenerative medicine aneurysm repair under compassionate use.

In Case 2, the patient passed away due to complications of MRSA infective endocarditis two months after his HAV implantation. While HAV conduits have been placed successfully in people with active infections [14], there was no indication of infection in this patient at the time of surgery. There were no sterility breaches noted intraoperatively. The patient did not develop a surgical site infection postoperatively over the next two months of follow-up. He was found to have an infection of his internal jugular HD catheter two months postoperatively, necessitating its removal. While we cannot know exactly, we hypothesize that his vascular reconstruction and related care helped his overall health while not protecting him from all future challenges.

Limitations of our case series include high-risk patient presentations and the intermediate timeline for outcomes. The patients in our study receiving HAV conduits under expanded access had multiple preexisting comorbidities at presentation and limited options for reconstruction due to their anatomical variance. Diseases unrelated to their operative courses led to mortality within one year in two of the three cases, limiting the ability to determine the intermediate and long-term efficacy of the HAV conduit in these patients. Another limitation is the small number of patients involved and the one-year follow-up interval as related to the surviving patient. The first HAV conduit construction under the EAP took place one year before this initial report, and the next two followed at bimonthly intervals. Future clinical research is warranted to determine the best indications for the use of this regenerative technology, including applications concerning aneurysm repair.

## Conclusions

This case series shares initial patient experiences in a nonprofit academic system of health in the field of regenerative medicine through the United States FDA’s EAP. Their care reality includes challenges endured by patients with ESRD, along with successful creation and intermediate-term HD access with HAV conduit, preventing complications such as infection, rejection, foreign body reactions, and aneurysm formation. Furthermore, this operative case series focuses on pioneering advanced regenerative medicine techniques in patients requiring aneurysm repairs. We anticipate investigating the expanded utility of HAV conduits in regenerative medicine to provide compassionate care for patients with ESRD, aneurysms, and other critical needs. However, additional long-term data will be needed for a future study.
